# Deflection Test of Wire-Integrated Steel Deck Plates with Various End Details

**DOI:** 10.3390/ma16062251

**Published:** 2023-03-10

**Authors:** Hye-Ji Lee, Keun-Hyeok Yang, Sanghee Kim, Jong-Kook Hong, Deung-Hwan Kim, Ju-Hyun Mun

**Affiliations:** 1 Department of Architectural Engineering, Kyonggi University, Suwon 16227, Republic of Koreayangkh@kgu.ac.kr (K.-H.Y.);; 2 School of Architecture, Sunchon National University, Jeonnam 57922, Republic of Korea; 3 Deck Business Division, Winhitech Co., Ltd., Seoul 08502, Republic of Korea

**Keywords:** deck plate, deflection, construction load, concrete self-weight, lattice foot, deflection limit

## Abstract

This experimental study was conducted to evaluate the deflection performance of wire-integrated steel deck plates with various end details subjected to cumulative gravity loads. In general, when deck plates are installed in the Republic of Korea, vertical bars are mounted at the ends of the wire-integrated deck plates. However, this process can extend the construction time, thus incurring additional costs. Consequently, this study aimed to examine the structural performance of a deck plate when a lattice foot—rather than a vertical bar—is installed at the end of it. A total of nine specimens were prepared; the experimental variables included the end details, height of the lattice truss girder, and structure type. To evaluate the deflection performance, the cumulative gravity load (as a construction load) and a concrete self-weight were applied to the specimens, and the deflections of each specimen were measured. In the experimental results, the deflection values of the specimen with vertical bars were 0.9~6.1 mm, while those for the specimen without vertical bars were 0.8~5.0 mm. This means that a lattice foot exhibits better deflection performance than conventional end details. Additionally, the deflection of the specimens satisfied the deflection limits required in the relevant standards.

## 1. Introduction

**Figure 1:**
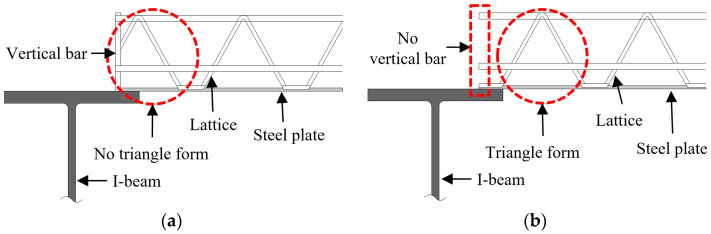
End details of the wire-integrated deck plate. (**a**) Conventional end details. (**b**) Proposed end details.

When wire-integrated deck plates are installed in the Republic of Korea, vertical bars are commonly welded to the top and bottom bars of the lattice truss girders because both parts of the lattice truss girders on wire-integrated steel deck plates conventionally have an open triangle form with a cut chord (Figure 1a). Therefore, the installation of a vertical bar at the end is required to ensure structural safety. However, this additional process can increase construction costs and cause delays. To address these problems, this study proposed a new end detail whereby lattice truss girders are cut at the lattice foot, which are then laid on the supports as shown in Figure 1b. From a structural perspective, a deck plate with a lattice foot at the end makes it possible for it to transfer the construction load and the concrete self-weight to support structures such as formwork for reinforced concrete (RC) beams or steel I-beams [1,2,3]. To stably transfer loads, deck plates should have enough flexural stiffness to limit a deflection at mid-span and to restrain slip at both ends [4]. Consequently, it is essential to verify that the deck plate’s lattice foot can transfer loads to the supports in a stable manner without the use of a vertical bar at the end.

In the Republic of Korea, research conducted on the deflection performance of wire-integrated deck plates has primarily consisted of deflection experiments using load bags/steel blocks, comparing the degree of deflection of each specimen under a construction load and/or concrete self-weight based on the deflection limits stipulated by the relevant standards [5,6]. The effect of the dynamic load [7] according to the pouring speed was not considered, and the load bag was used to load the load step-by-step. Kim et al. [8] performed the deflection test of the deck plate with variables, namely, the diameter of the lattice bar and the end details with a new buttress plate. Meanwhile, Lee et al. [9] evaluated the new deck plate system. Moreover, Kang and Kim [10] studied new end details for replacing vertical bars with a shear connector as a new technology. These studies reported that the deflection values of deck plates with various end details were similar, and the studies were satisfied with the limitation of deflection in codes [8,9,10,11]. However, studies concerning the presence/absence of vertical bars and the triangle form of lattices are still lacking.

Outside the Republic of Korea, studies on deck plates have focused on evaluating the behavior of composite deck slabs after the placement of concrete rather than the behavior of deck plates during construction. Most studies on composite deck slabs have evaluated the slabs’ structural performance and fire resistance [12,13,14,15,16]. Additionally, the use of composite deck slabs in bridges has been more actively researched than that in buildings [17,18,19,20]. To summarize, research is lacking on deflection tests on wire-integrated steel deck plates without vertical bars considering the construction load and concrete self-weight together.

Consequently, this experimental study aimed to examine the deflection performance of wire-integrated deck plates with various end details subjected to cumulative gravity loads. Additionally, it compared the deflection performance of specimens with the proposed end detail with that of specimens with the conventional end detail and determined whether the specimens with various end details satisfied the deflection limits required by the relevant standards.

## 2. Design Standards for Deck Plates during Construction

### 2.1. Design Loads

#### 2.1.1. KDS 21 50 00 [21]

Dead and construction loads as well as gravity loads can be used in the design of formwork and shore scaffolds in KDS 21 50 00 [21]. The dead load during the construction stage is the sum of the weights of the reinforced concrete and formwork, with the unit weight of RC being 24 kN/m^3^ in the case of normal concrete, 20 kN/m^3^ in the case of Type 1 lightweight concrete, and 17 kN/m^3^ in the case of Type 2 lightweight concrete. At least 0.4 kN/m^2^ is used for the weight of the formwork. The construction load is assigned to be at least 2.5 kN/m^2^ per horizontally projected area when the height of the poured concrete is less than 0.5 m, 3.5 kN/m^2^ when it is at least 0.5 m but less than 1.0 m, and 5.0 kN/m^2^ when it is 1.0 m or more. For the gravity load, that is, the sum of the dead load and construction load, a minimum of 5.0 kN/m^2^ is applied regardless of the height of the poured concrete.

#### 2.1.2. SDI C-2017 [22]

The SDI C-2017 [22] standard presents load combinations for the *allowable stress design* (ASD) or *load and resistance factor design* (LRFD), respectively. The strength and deflection of the deck slab can be evaluated based on the load combinations, which, for ASD, are as follows:

(1) $$w_{dc}+w_{dd}+w_{lc}$$

(2) $$w_{dc}+w_{dd}+P_{lc}$$

(3) $$w_{dd}+w_{cdl}$$

The load combinations for the LRFD are as follows:

(4) $$1.6w_{dc}+1.2w_{dd}+1.4w_{lc}$$

(5) $$1.6w_{dc}+1.2w_{dd}+1.4P_{lc}$$

(6) $$1.2w_{dd}+1.4w_{cdl}$$

where *w_dc_* is the concrete self-weight, *w_dd_* is the self-weight of the deck plate, *w_lc_* is the uniformly distributed construction live load including fresh concrete (1.0 kN/m^2^ or more is used; however, if the concrete is being transported or placed with equipment, or heavier motorized finishing equipment is being used, then 2.5 kN/m^2^ or more is used), *P_lc_* is the concentrated construction live load per unit width (1 m) of the deck plate (2.2 kN or more is used), and *w_cdl_* is the uniformly distributed construction load including the deck plate (2.5 kN/m^2^ or more is used).

### 2.2. Deflection Limits

#### 2.2.1. Composite Deck Slab Standard (Proposal) and Commentary [23]

The Composite Deck Slab Standard (Proposal) and Commentary [23]—hereafter referred to as the CDSS-98 standard—stipulates that the concrete self-weight and construction load (1.5 kN/m^2^) should be used to calculate the deck deflection, which is limited to 1/180 of the clear span, or 19 mm or less. Here, the extent of the deck-plate deflection can be calculated as the deflection relative to the support point.

#### 2.2.2. SDI C-2017 [22] and BS EN 1994-1-1 [24]

When calculating deflection, the construction load is considered a temporary load, and the deck plate is assumed to be in an elastic state under the construction load. Consequently, when calculating the deck deflection, only the self-weights of the concrete and deck plate are used. The deflection limit indicated in the SDI C-2017 [22] standard is 1/180 of the clear span, or 19 mm or less, and that indicated in the BS EN 1994-1-1 [24] standard is 1/180 of the clear span, or 20 mm or less.

#### 2.2.3. BS 5950-4 [25]

When calculating the deflection of the deck plate, the standard indicates that only the dead load (concrete and deck-plate self-weights) should be used, as is the case with the SDI C-2017 [22] and BS EN 1994-1-1 [24] standards. The deflection limits are classified based on whether they consider the ponding effect or not. When the ponding effect is not considered, the deflection caused by the dead load is limited to 1/180 of the clear span, or 20 mm or less; if the ponding effect it is considered, the deflection caused by dead load is limited to 1/130 of the clear span, or 30 mm or less. Moreover, if the extent of deflection in the deck exceeds 1/10 of the thickness of the composite deck slab, it is necessary to include the additional self-weight of the concrete due to the deck ponding effect in the slab self-weight.

## 3. Experimental Program

### 3.1. Test Specimens

**Figure 2:**
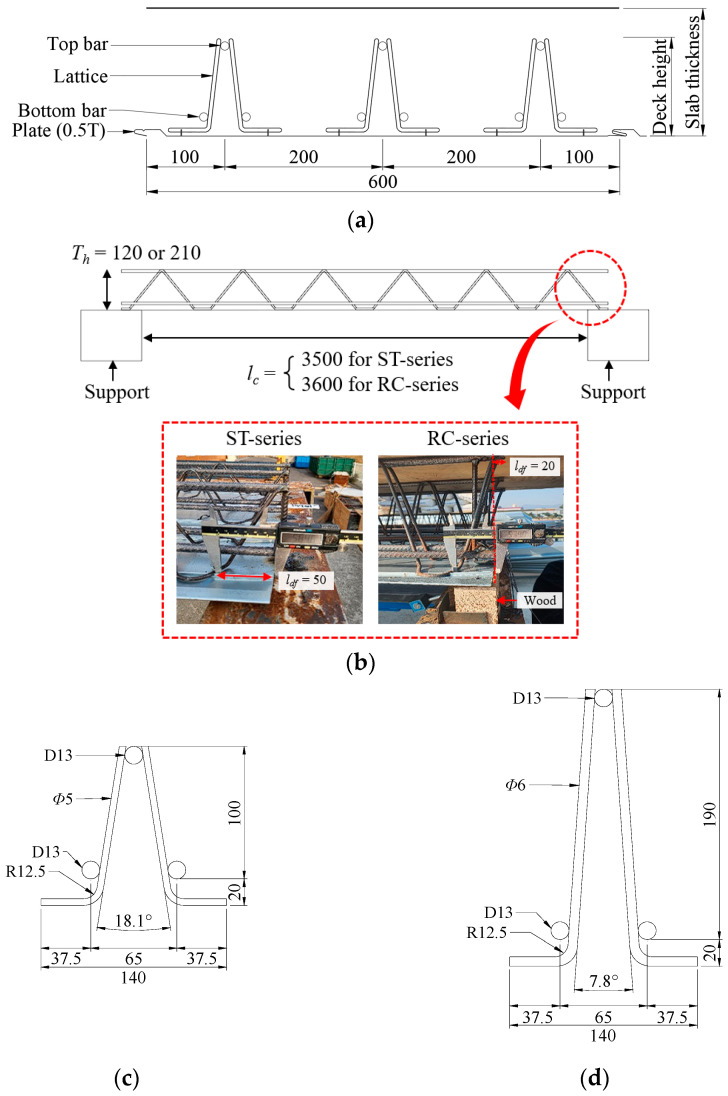
Details of each lattice truss girder (units in mm). (**a**) Section of wire-integrated steel deck plate. (**b**) Size details of wire-integrated steel deck plate. (**c**) Lattice truss girder of 120 mm. (**d**) Lattice truss girder of 210 mm.

**Figure 3:**
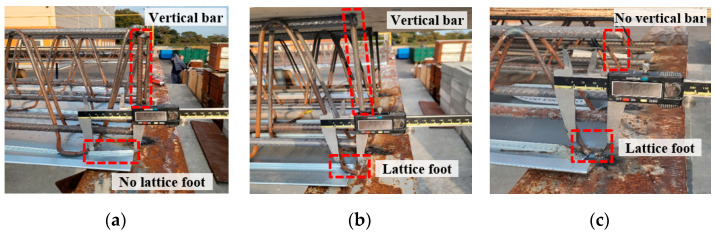
End details of test specimens. (**a**) End detail A. (**b**) End detail B. (**c**) End detail C.

A total of nine experimental specimens were prepared for the evaluation of the deflection performance of the wire-integrated deck plates with various end details based on the cumulative gravity loads, as shown in Table 1 [26]. The major variables were the end details, deck plate height, and structure type. The width of the deck plate was set to 600 mm (unit width) by arranging three truss rows (Figure 2a). The total length of the deck plate was classified based on the structure type, that is, steel and RC structures, with the lengths of these being 3600 mm and 3740 mm, respectively. The length of the foot at the end of the deck plate was 50 mm for the steel structure and 20 mm for the RC structure (Figure 2b). Composite deck slabs of 150 mm and 250 mm used lattice truss girders of 120 mm and 210 mm, respectively. Figure 2c shows details of a lattice truss girder of 120 mm that uses a 5 mm diameter bar for the lattice and 13 mm diameter bars for the top and bottom main bars. Figure 2d shows details of a lattice truss girder of 210 mm that uses a 6 mm diameter bar for the lattice and 13 mm diameter bars for the top and bottom main bars. Moreover, a 0.5 mm hot-dipped zinc-coated steel sheet was used to fabricate the deck plates. Figure 3 shows the various end details. *End Detail A* is a conventional detail having vertical bars but no lattice foot, *End Detail B* has a lattice foot and vertical bar together, and *End Detail C* has only a lattice foot.

### 3.2. Loading and Measurement Details

**Figure 4:**
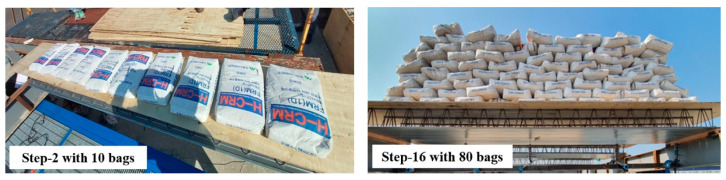
Photos at *Step-2* with 10 load bags and *Step-16* with 80 load bags.

The construction load and concrete self-weight used to examine the structural performance of the deck plates according to load step were calculated based on the KDS 21 50 00 [21] standard. Table 2 presents the applied load calculated using the design standards based on slab thickness. Dead load (concrete self-weight) was computed by the unit weight of concrete multiplied by the height of the deck plates. Note that the deck plate self-weight including lattice truss girder and plate was not considered, as the camber was applied in consideration of that. Regarding Figure 4, only wire-integrated steel deck plates were laid on both supports, and then the camber was applied. The load bags were consequently stacked. The maximum loads per unit area for slab thicknesses of 150 mm and 250 mm were 6.1 kN/m^2^ and 8.5 kN/m^2^, respectively. To apply loads with a uniform distribution to the specimens, wooden plates were placed over the deck plates before the load bags were stacked. For loading, a 25 kg load bag was used, with a total of 125 kg of load bags stacked on a specimen at each loading step (Table 3). In Table 3, the load per unit area is the total weight divided by the net area of the wire-integrate steel deck plate in each step. Figure 4 displays images captured at Step-2 (with 10 load bags stacked on the specimen) and Step-16 (with 80 load bags).

**Figure 5:**
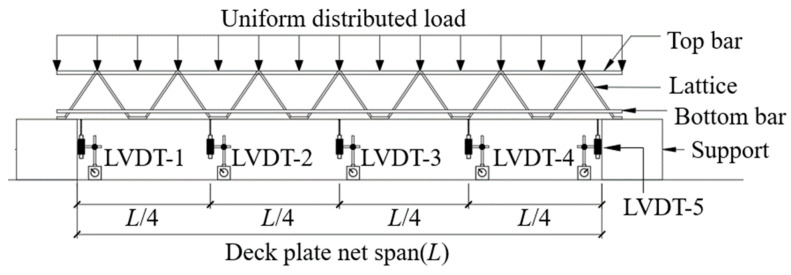
Locations of LVDTs.

To measure the specimen deflection, 5 linear variable displacement transducers (LVDTs) with a capacity of 50 mm were installed at 5 points, as shown in Figure 5. Moreover, the LVDT installation locations were at the center of the deck plate along its width.

## 4. Experimental Results and Discussion

### 4.1. Load Case for Deflection Evaluation

A total of four load cases (LCs) were considered to evaluate the deflection performance of the wire-integrated deck plate. In *LC-1*, the construction live load of 1.0 kN/m^2^ was determined without considering the use of concrete transportation and placement with equipment or the use of heavier motorized finishing equipment, in accordance with the SDI C-2017 [22] standard. In *LC-2*, the construction live load of 2.5 kN/m^2^ was determined while considering concrete transportation and placement with equipment and the use of heavier motorized finishing equipment, in accordance with the KDS 21 50 00 [21] and SDI C-2017 [22] standards. In *LC-3*, the load was determined based on a reinforced concrete unit weight of 24 kN/m^3^ in accordance with the KDS 21 50 00 [21] standard. In *LC-4*, the load was determined as the total load considering the working load and the concrete self-weight. A camber was applied to all specimens; consequently, the deck plate self-weight was not considered in all LCs. As such, the load per unit area based on the slab thickness is as shown in Table 4.

### 4.2. Deck Plate Load-Deflection Relationship

The results of the relative downward deflection at the center of each specimen based on the loading step are summarized in Table 5. Here, the relative downward deflection is determined with the deflection difference of the center deflection (*LVDT-3*) less the average of the deflection values at the end points (*LVDT-1*, *LVDT-5*), without considering the camber height.

**Figure 6:**
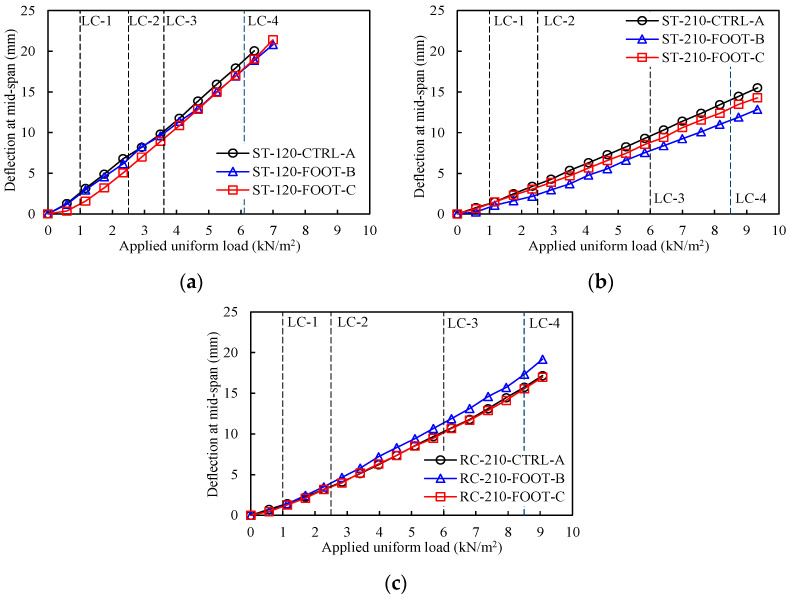
Applied load–deflection response. (**a**) Group-1. (**b**) Group-2. (**c**) Group-3.

The relationships between the applied load per unit area and the relative deflection at the center are shown in Figure 6. Overall, the load–deflection relationships of specimens in the same group were similar regardless of the presence/absence of a vertical bar or lattice foot. Comparing the experimental results in detail, ST-120-Foot-No in Group-1 with *End Detail C* had better deflection performance up to *Step-9* than the other specimens. In Group-2, ST-210-FOOT-B had the best deflection performance at all loading steps, and the deflection performance of ST-210-FOOT-C was better than that of ST-210-CTRL-A. In Group-3, the load–deflection relationships of RC-210-CTRL-A and RC-210-FOOT-C were similar, both being slightly better than that of RC-210-FOOT-B.

**Figure 7:**
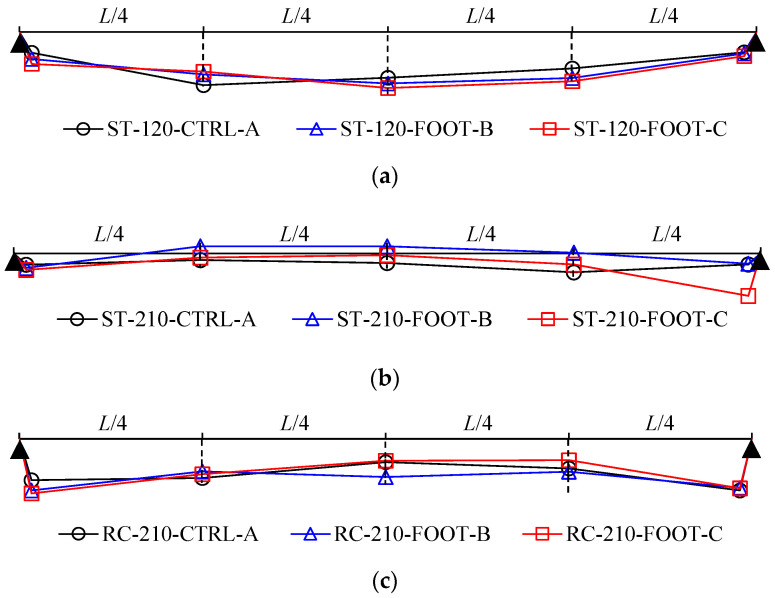
Deflection distributions at the final loading step. (**a**) Group-1. (**b**) Group-2. (**c**) Group-3.

The deflection distributions of the specimens in each group are as shown in Figure 7; they were similar for all specimens regardless of the deck plate type and height of the lattice truss girder. During the final loading step, there was a deflection average of 6.73 mm at the points on both ends of RC-210-CTRL-A, RC-210-FOOT-B, and RC-210-FOOT-C, that is, the RC beam point specimens; this was approximately 2.37 times higher than that of the steel beam point specimens. It can be assumed to have been caused by shrinkage deformation in the wood.

### 4.3. Evaluation of Allowable Deck Plate Deflection

The measured deflection of the specimens under dead and construction loads was compared with the allowable deflection calculated based on the CDSS-98 [23] and SDI C-2017 [22] standards, as shown in Table 6. The allowable deflection indicated in the two standards is the minimum of 1/180 of the clear span, or 19 mm. The clear spans of the specimens used in this study were 3500 mm and 3600 mm for steel and RC structural types, respectively; consequently, the allowable deflection in this experimental study was 19 mm. The measurement of a deflection was initiated in the cambered state, with the uplift of the camber being considered when deciding the permissible deflection.

The maximum deflection, sum of the upward camber, and downward deflection by load bags of the ST-120-CTRL-A, ST-120-FOOT-B, and ST-120-FOOT-C specimens were 6.1 mm, 4.9 mm, and 5.0 mm, respectively, and all values satisfied the allowable deflection standard of 19 mm. The maximum deflection values of the ST-210-CTRL-A, ST-210-FOOT-B, and ST-210-FOOT-C specimens were 0.5 mm, −2.1 mm, and −0.5 mm, respectively, which also satisfied the allowable deflection standard of 19 mm. The maximum deflection values of the RC-210-CTRL-A, RC-210-FOOT-B, and RC-210-FOOT-C specimens were 0.9 mm, 2.5 mm, and 0.8 mm, respectively, which again satisfied the allowable deflection standard. Consequently, the deflections of all specimens with camber satisfied the allowable deflection stipulated by the CDSS-98 [23] and SDI C-2017 [22] standards.

### 4.4. Flexural Stiffness

**Figure 8:**
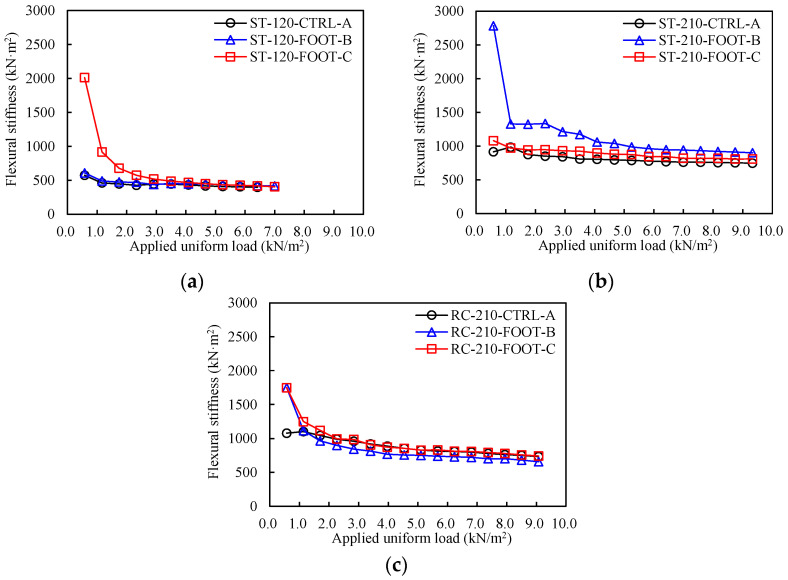
Flexural stiffness based on loading steps. (**a**) Group-1. (**b**) Group-2. (**c**) Group-3.

The flexural stiffness (*EI*) of the specimens could be calculated from the applied load and the center relative deflection using the following equation, assuming that the specimens were a simple beam:

(7) $$EI=\frac{5wl_{c}{}^{4}}{384\delta }$$

where *w* is the uniformly distributed load applied to the specimens, *l_c_* is the clear span, and δ is the value of the relative deflection at the center. The flexural stiffness of specimens at each loading step is shown in Figure 8, classified into Groups 1–3. In Group 1, the *EI* of ST-120-FOOT-C is higher until *Step-6*. From *Step-7*, the *EIs* of the three specimens are similar. In Group 2, the *EI* of ST-210-FOOT-B is higher than that of the other two specimens until the end of the experiments, with the *EI* of ST-210-CTRL-A and ST-210-FOOT-C being similar. In Group 3, the *EIs* of RC-210-FOOT-B and RC-210-FOOT-C are higher than that of RC-210-CTRL-A only during the first loading in *Step-1*. From *Step-3*, the *EI* of RC-210-FOOT-B is somewhat lower than that of the other two specimens. Overall, the *EI* of the deck plate with a lattice foot is similar to or greater than that of the conventional deck plate regardless of the experimental variables.

## 5. Conclusions

This study performed deflection tests on wire-integrated deck plate subjects, gradually increasing the gravity load to evaluate their deflection performance. The variables of this experimental study were three end details, the height of the lattice truss girder, and two structural types that are reinforced concrete and steel structures. The conclusions obtained from the experimental results are as follows:

1. The three types of end detail included the use of a conventional detail with a vertical bar, a vertical bar and a lattice foot, or only a lattice foot. Regardless of the end details, specimens of the same structural type and lattice truss girder height had similar deflection diagrams, with each specimen exhibiting a similar degree of deflection and flexural stiffness. That is, even when the wire-integrated deck plate had only a lattice foot with no end vertical bar, its performance was equivalent to that of a wire-integrated deck plate with conventional detail.
2. All specimens satisfied the deflection limits (19 mm) indicated in the Composite Deck Slab Standard (Proposal) and Commentary [15] and SDI C-2017 [14] standards. Consequently, the specimen with just the lattice foot could be used in construction projects.
3. In summary, specimens with lattice foot details exhibited similar or higher flexural stiffness than specimens with conventional end details, that is, the lattice foot could transfer gravity loads from wire-integrated deck plates to the supporting structures and could replace vertical bars in practical applications. Therefore, it is considered that a new end detail is used in the construction field, allowing the construction time and cost to be reduced.

## Figures and Tables

**Table 1:** Details of the test specimens.

Group	Specimen ID	End Details ^1^	Structure Type	Truss Girder Height (*T_h_*) [mm]	Slab Thickness [mm]	Deck Length [mm]	Deck Clear Span (*l_c_*) [mm]	Deck Foot Length (*l_df_*) [mm]	Camber [mm]	Top Bar / Bottom Bar	Lattice Bar [mm]
1	ST-120-CTRL-A	A	Steel	120	150	3600	3500	50	14.0	3-HD13 / 6-HD13	*Φ*5
	ST-120-FOOT-B	B									
	ST-120-FOOT-C	C									
2	ST-210-CTRL-A	A		210	250						*Φ*6
	ST-210-FOOT-B	B									
	ST-210-FOOT-C	C									
3	RC-210-CTRL-A	A	RC	210	250	3740	3600	20	14.8		
	RC-210-FOOT-B	B									
	RC-210-FOOT-C	C									

^1^ End details are shown in Figure 3.

**Table 2:** Applied loads.

Slab Thickness [mm]	Construction Load [kN/m^2^]	Dead Load (Concrete) [kN/m^2^]	Total Load [kN/m^2^]
150	2.5	3.6	6.1
250		6.0	8.5

**Table 3:** Loading plan.

Loading Step	Number of Load Bags	Total Weight of Load Bags [kg]	Load per Unit Area [kN/m^2^]	
			Steel Beam	RC Beam
*Step-1*	5	125	0.58	0.57
*Step-2*	10	250	1.17	1.13
*Step-3*	15	375	1.75	1.70
*Step-4*	20	500	2.33	2.27
*Step-5*	25	625	2.92	2.84
*Step-6*	30	750	3.50	3.40
*Step-7*	35	875	4.08	3.97
*Step-8*	40	1000	4.67	4.54
*Step-9*	45	1125	5.25	5.10
*Step-10*	50	1250	5.83	5.67
*Step-11*	55	1375	6.42	6.24
*Step-12*	60	1500	7.00	6.81
*Step-13*	65	1625	7.58	7.37
*Step-14*	70	1750	8.17	7.94
*Step-15*	75	1875	8.75	8.51
*Step-16*	80	2000	9.33	9.07

**Table 4:** Load case based on slab thickness.

Load Cases	Load	Load Per Square Meter [kN/m^2^]	
		Slab Thickness 150 mm (Lattice Truss Girder 120 mm)	Slab Thickness 250 mm (Lattice Truss Girder 210 mm)
*LC-1*	*w_lc_*	1.0	1.0
*LC-2*	*w_lc_*	2.5	2.5
*LC-3*	*w_dc_*	3.6	6.0
*LC-4*	*w_dc_ + w_dd_*	6.1	8.5

**Table 5:** Summary of test results.

Group	Specimen ID	Deflection at Mid-Span [mm]															
		*Step-1*	*Step-2*	*Step-3*	*Step-4*	*Step-5*	*Step-6*	*Step-7*	*Step-8*	*Step-9*	*Step-10*	*Step-11*	*Step-12*	*Step-13*	*Step-14*	*Step-15*	*Step-16*
	Load per Unit Area [kN/m^2^]	0.58	1.17	1.75	2.33	2.92	3.50	4.08	4.67	5.25	5.83	6.42	7.00	7.58	8.17	8.75	9.33
1	ST-120-CTRL-A	1.26	3.14	4.88	6.79	8.19	9.82	11.77	13.87	15.95	17.95	20.07	-	-	-	-	-
	ST-120-FOOT-B	1.19	2.95	4.57	6.18	8.27	9.60	11.37	12.94	15.03	16.95	18.89	20.82	-	-	-	-
	ST-120-FOOT-C	0.36	1.58	3.21	5.05	7.00	8.93	10.87	12.87	14.96	17.00	19.03	21.43	-	-	-	-
2	ST-210-CTRL-A	0.79	1.47	2.48	3.40	4.29	5.37	6.30	7.28	8.27	9.30	10.33	11.42	12.39	13.44	14.46	15.51
	ST-210-FOOT-B	0.26	1.09	1.64	2.17	2.98	3.70	4.78	5.57	6.60	7.54	8.41	9.24	10.09	11.01	11.91	12.86
	ST-210-FOOT-C	0.67	1.49	2.30	3.06	3.89	4.70	5.65	6.59	7.46	8.55	9.42	10.62	11.54	12.39	13.47	14.28
	Load per Unit Area [kN/m^2^]	0.57	1.13	1.70	2.27	2.84	3.40	3.97	4.54	5.10	5.67	6.24	6.81	7.37	7.94	8.51	9.07
3	RC-210-CTRL-A	0.73	1.43	2.26	3.19	4.10	5.14	6.21	7.37	8.55	9.68	10.73	11.80	13.11	14.45	15.74	17.17
	RC-210-FOOT-B	0.45	1.41	2.45	3.50	4.67	5.80	7.19	8.32	9.43	10.66	11.87	13.12	14.59	15.72	17.33	19.19
	RC-210-FOOT-C	0.45	1.26	2.11	3.17	3.99	5.20	6.31	7.37	8.54	9.48	10.67	11.70	12.88	14.14	15.55	16.98

**Table 6:** Summary of deflection evaluation.

Group	Specimen ID	Camber [mm] (1)	Measurement Deflection [mm]		Maximum Deflection [mm] ((1) + (2))	Decision	
			*LC-3*	*LC-4* (2)		Allowable Deflection [mm]	OK/NG
1	ST-120-CTRL-A	−14.0	9.8	20.1	6.1	19.0	OK
	ST-120-FOOT-B		9.6	18.9	4.9		OK
	ST-120-FOOT-C		8.9	19.0	5.0		OK
2	ST-210-CTRL-A		10.3	14.5	0.5		OK
	ST-210-FOOT-B		8.4	11.9	−2.1		OK
	ST-210-FOOT-C		9.4	13.5	−0.5		OK
3	RC-210-CTRL-A	−14.8	10.7	15.7	0.9		OK
	RC-210-FOOT-B		11.9	17.3	2.5		OK
	RC-210-FOOT-C		10.7	15.6	0.8		OK

## Data Availability

Not applicable.

## References

[1] Shin J., Lee J., Lee Y., Kim B. (2019). Experimental and numerical investigation on structural performance of steel deck plate bolted with truss girder. Appl. Sci..

[2] Lee Y.J. (2010). Test research of structural safety for steel wire-integrated deck plate system. KSMI.

[3] Choi I.R., Lee G.R., Jeon S.H., Kyung J.H. (2021). Deflection performance evaluation of new deep deck with 300 mm depth during construction loads. J. Korean Soc. Steel Const..

[4] Kim S.H., Hong J.K., Kim D.H. (2023). Analytical study on structural performance of wire-integrated steel decks with varied lattice end-support configurations. KSMI.

[5] Heo I.W., Han S.J., Choi S.H., Kim K.S., Kim S.B. (2019). Experimental study on structural behavior of double ribbed deep-deck plate under construction loads. J. Korea Inst. Struct. Maint. Insp..

[6] Jeon S.H., Kyung J.H., Kim Y.H., Choi S.M., Yang I.S. (2015). Deflection evaluation of the constructing-load carrying capacity for deep decking floor system reinforced with both ends cap plates. J. Korean Soc. Steel Const..

[7] Civalek Ö., Öztürk B. (2009). Discrete singular convolution algorithm for non-linear transient response of circular plates resting on Winkler-Pasternak elastic foundations with different types of dynamic loading. Indian J. Eng. Mater. Sci..

[8] Kim S.B., Kang M.J., Hwang B.C., Kim S.S. (2014). Structural performance evaluation for steel wire-integrated deck plate according to the diameter of the lattice bar. J. Korean Soc. Hazard Mitig..

[9] Lee J.E., Kim B.Y., Jung B.J. (2014). Evaluation of structural safety and economic feasibility for removable steel plate eco deck plate. JAIK.

[10] Kang M.J., Kim S.S. (2015). Structural performance evaluation of steel wire-integrated deck plate according to the construction load. JAIK.

[11] Yoo B.U. (2006). A study on structural behavior of composite deck plate using a pre-assembled re-bar truss. KSMI.

[12] Liu R., Yang Y., Zhou X. (2018). Experimental study on fatigue performance of composite beam with steel-plate-concrete composite decks. Constr. Build Mater..

[13] Xiang D., Liu Y., Shi Y., Xu X. (2022). Vertical shear capacity of steel-concrete composite deck slabs with steel ribs. Eng. Struct..

[14] Zhao H., Zhao J., Wang R., Zhang W., Liu F., Wu S. (2022). Thermal behavior of composite slabs with closed profiled steel decking and recycled aggregate concrete in fire. Fire Saf. J..

[15] Seo J., Hatfield G., Kimn J.H. (2016). Probabilistic structural integrity evaluation of a highway steel bridge under unknown trucks. J. Struct. Integr. Maint..

[16] Bu Y., Li M., Wei C., Cheng Z., Cui C., Bao Y. (2023). Experimental and analytical studies on flexural behavior of composite bridge decks with orthotropic steel deck and Ultra-High-Performance Concrete (UHPC) slab under negative moment. Eng. Struct..

[17] Cheng J., Xu M., Xu H. (2019). Mechanical performance analysis and parametric study of double-deck plate-truss composite steel girders of a three-tower four-span suspension bridge. Eng. Struct..

[18] Sebastian W.M. (2021). Bi-Axial behaviours of abraded and rehabilitated FRP decks as anisotropic plates under concentrated wheel loading. Eng. Struct..

[19] Colombani I.A., Andrawes B. (2022). A Study of multi-target image-based displacement measurement approach for field testing of bridges. J. Struct. Integr. Maint..

[20] Moomen M., Siddiqui C. (2022). Probabilistic deterioration modeling of bridge component condition with random effects. J. Struct. Integr. Maint..

[21] (2022). Design Standard of Formwork and Support Bar.

[22] (2017). Composite Steel Floor Deck-Slabs.

[23] AIK (1998). Composite Deck Slab Standard (Proposal) and Commentary.

[24] (2004). Design of Composite Steel and Concrete Structures General Rules and Rules for Buildings.

[25] (1994). Structural Use of Steelwork in Building-Part 4: Code of Practice for Design of Composite Slabs with Profiled Steel Sheeting.

[26] Yang K.H., Hong J.K., Kim S.H., Lee H.J. (2022). Structural Performance Evaluation and Design Guideline for End Supports of EXTRA Deck Plate System.

